# Effects of Cabozantinib, a small molecule tyrosine kinase inhibitor, on the immune permissiveness of the tumor microenvironment and immune-mediated killing of tumor cells

**DOI:** 10.1186/2051-1426-2-S3-P185

**Published:** 2014-11-06

**Authors:** Anna R Kwilas, Andressa Ardiani, Renee N Donahue, Dana Aftab, James W Hodge

**Affiliations:** 1 NCI/CCR/LTIB, Bethesda, MD, USA; 2 Exelixis Inc., South San Francisco, CA, USA

## 

Combination therapy for the treatment of cancer is becoming increasingly important as the complexity of carcinogenesis and the availability of targeted therapies continues to expand. Successful combination therapy would target the primary tumor and distant metastases from multiple different angles to ensure a higher probability of cancer eradication. Immunotherapy is quickly gaining acceptance as an active therapeutic modality in a range of cancers, however cancer is generally thought of as an immunosuppressive disease and often it may be difficult to induce a successful antitumor immune response. Thus, combining cancer immunotherapy with other agents that have immunomodulatory capabilities could significantly improve its efficacy. We have evaluated the receptor tyrosine kinase inhibitor Cabozantinib for its ability to modulate the immune system as well as alter the phenotype of tumor cells to determine if a rationale exists for combining this targeted agent with cancer immunotherapy. Cabozantinib inhibits multiple receptor tyrosine kinases including MET, which has been shown to reduce dendritic cell function, and VEGFR2, which plays a major role in tumor vascularization thus affecting immune infiltration. Our studies indicated that Cabozantinib was able to alter the phenotype of MC38-CEA murine colon carcinoma cells, increasing their sensitivity to T cell-mediated killing. Cabozantinib treatment also modified the composition of the peripheral immune compartment, increasing the frequency of effector T cells while simultaneously reducing the frequency of suppressor cells. This alteration translated to the tumor microenvironment resulting in the generation of a more permissive immune environment. When combined with a poxviral-based cancer vaccine targeting a self-antigen, rMVA/F-CEA/TRICOM, Cabozantinib treatment also resulted in significantly reduced function of regulatory T cells and increased cytokine production from effector T cells in response to the antigen. In the MC38-CEA tumor model, Cabozantinib treatment alone significantly reduced tumor growth rate but was unable to induce complete and durable tumor regression at a dose of 10 mg/kg/day. However, the alterations to the immune landscape as well as the direct modification of the tumor cells led to markedly improved antitumor activity when Cabozantinib was combined with rMVA/F-CEA/TRICOM, resulting in complete and durable tumor regression in 50% of tumor-bearing mice. These data provide a scientific rationale for the clinical combination of Cabozantinib with immunotherapy for the treatment of cancer patients.

